# Two new species of *Ooceraea* (Hymenoptera, Formicidae, Dorylinae) from India with ten-segmented antennae

**DOI:** 10.3897/zookeys.1010.58436

**Published:** 2021-01-13

**Authors:** Himender Bharti, Joginder Singh Rilta, Tarun Dhadwal

**Affiliations:** 1 Department of Zoology & Environmental Sciences, Punjabi University, Patiala, Punjab, 147002, India Punjabi University Patiala India

**Keywords:** *
Cerapachys
*, distribution, illustrated key, *O. decamera* sp. nov., *O. joshii* sp. nov., systematics, taxonomy

## Abstract

Two new species, *O. decamera***sp. nov.** and *O. joshii***sp. nov.**, of the ant genus *Ooceraea* are described from India. These species differ from other known congeners on the basis of number of antennal segments. An illustrated key to the known species based on the worker caste is also provided.

## Introduction

The taxonomic history of the ant genus *Ooceraea* Roger, 1862 has been challenging, since its inception based on the type species *O. fragosa*. The taxonomic ambiguity has led to its uncertain placements in different subfamilies: in Myrmicinae (Mayr 1865; Emery 1877), in Ponerinae (Dalla Torre 1893; Forel 1893) and in Dorylinae (Emery 1895). *Ooceraea* was treated as a subgenus of *Cerapachys* (Emery 1902; Wheeler W.M. 1902; Emery 1911), and as a junior synonym of *Cerapachys* (Brown 1975). This dilemma has lately been resolved with a comprehensive revision of generic-level classification of the subfamily Dorylinae. *Ooceraea* was resurrected as a valid genus in Dorylinae with a distinctive combination of characters, by which it can be distinguished from other Dorylinae genera. These include: propodeal spiracle positioned low on the sclerite; pygidium armed with modified setae; antennae with 11 or fewer segments; pronotomesopleural suture developed; abdominal segment III strongly tubulated (forming “postpetiole”) and no constrictions between abdominal segments IV, V and VI. *Ooceraea* can be distinguished from the closely allied *Syscia* Roger, 1861 on the basis of abdominal segment III relatively narrow in dorsal view and similar in size to the preceding abdominal segment II (petiole); in lateral view, abdominal tergite IV not folding over sternite and the anterior portion of the sternite visible; hind basitarsi not dilating distally, circular in cross-section and metabasitarsal glands absent (Borowiec 2016).

The genus is currently represented by 14 species (Bolton 2020). Six of these are reported from the Australian and Oceanian regions, five from the Oriental region and two species from the Palearctic region (Holt et al. 2013; Janicki et al. 2016; Guénard et al. 2017; Yamada et al. 2018; Zhou et al. 2020); while the 14^th^ species *O. biroi* (Forel, 1907) is probably native to the Asian continent, and has been introduced to Southeast Asia, the Pacific islands, Madagascar and the Caribbean islands (Borowiec 2016; Janicki et al. 2016; Guénard et al. 2017) (Fig. 10). The antennal count has been found to be one of the significant species-level diagnostic characters in the genus. Eight of the known *Ooceraea* species possess nine-segmented antennae, while five possess eleven- segmented antennae and one species has recently been reported with eight-segmented antennae (Zhou et al. 2020). In India, the genus is represented by two species viz. *Ooceraea alii* (Bharti & Akbar, 2013) and *Ooceraea besucheti* (Brown, 1975) with nine- and eleven-segmented antennae respectively (Bharti et al. 2016). Here in, we describe two new species with ten-segmented antennae from India, thus adding to the known diversity of this considered rare genus. A key to the known species based on the worker caste is also provided.

## Materials and methods

**Figure 1.**
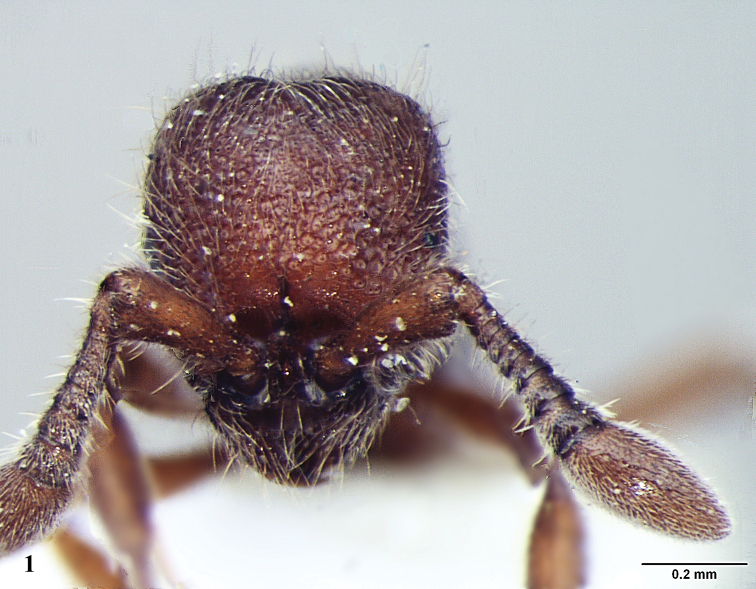
*Ooceraea joshii* sp. nov. Head in full-face view.

**Figure 2.**
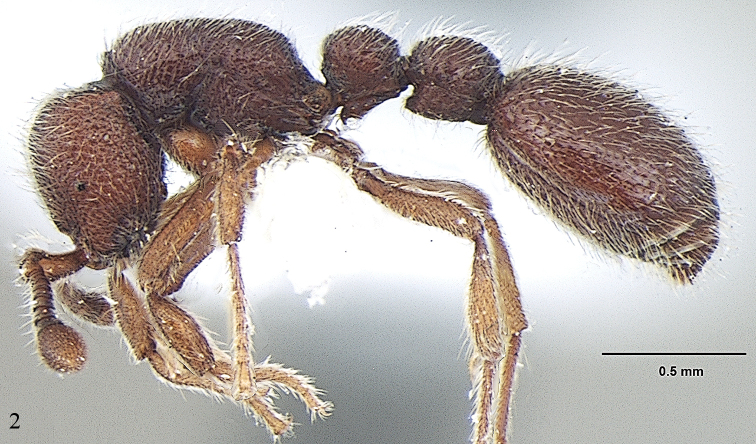
*Ooceraea joshii* sp. nov. Body in profile view.

**Figure 3.**
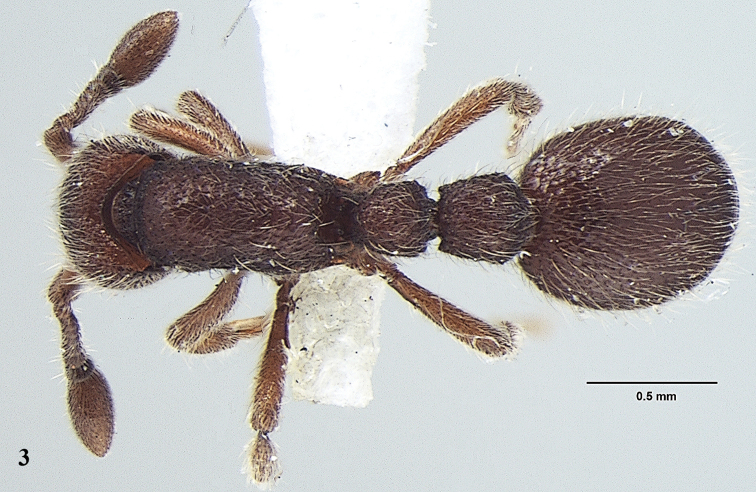
*Ooceraea joshii* sp. nov. Body in dorsal view.

**Figure 4.**
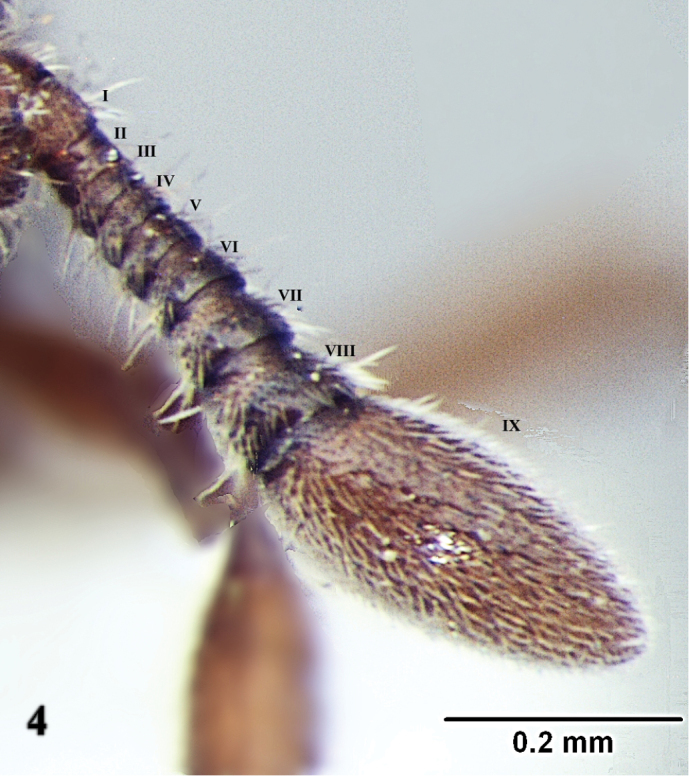
*Ooceraea joshii* sp. nov. Funicular segments.

**Figure 5.**
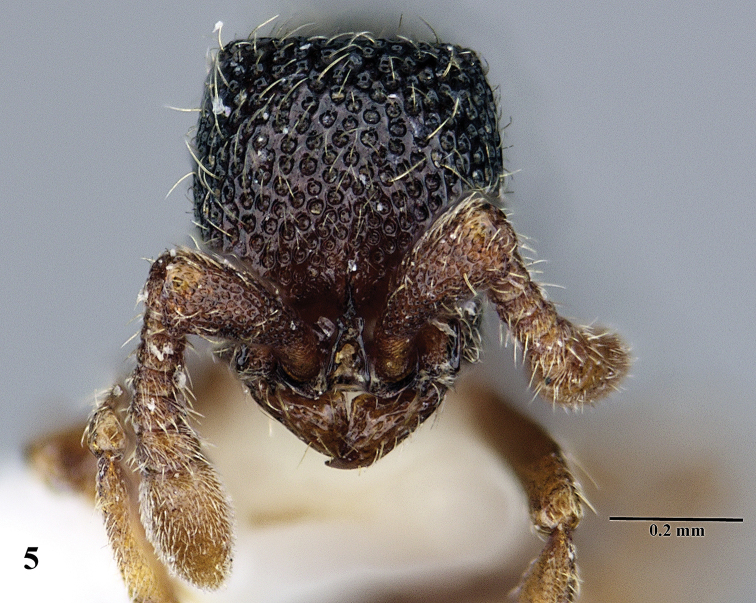
*Ooceraea decamera* sp. nov. Head in full-face view.

**Figure 6.**
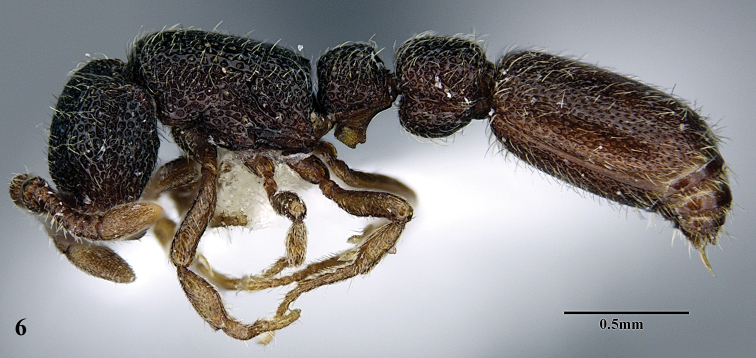
*Ooceraea decamera* sp. nov. Body in profile view.

**Figure 7.**
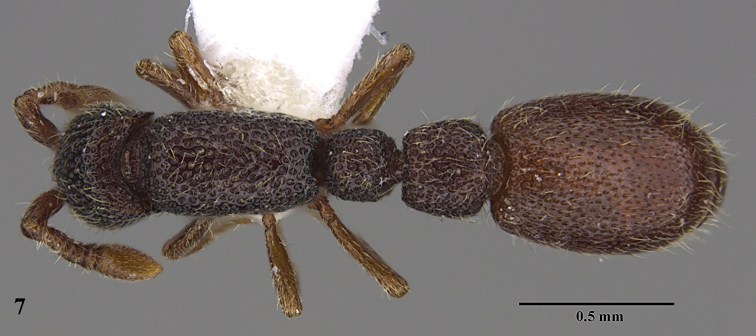
*Ooceraea decamera* sp. nov. Body in dorsal view.

**Figure 8.**
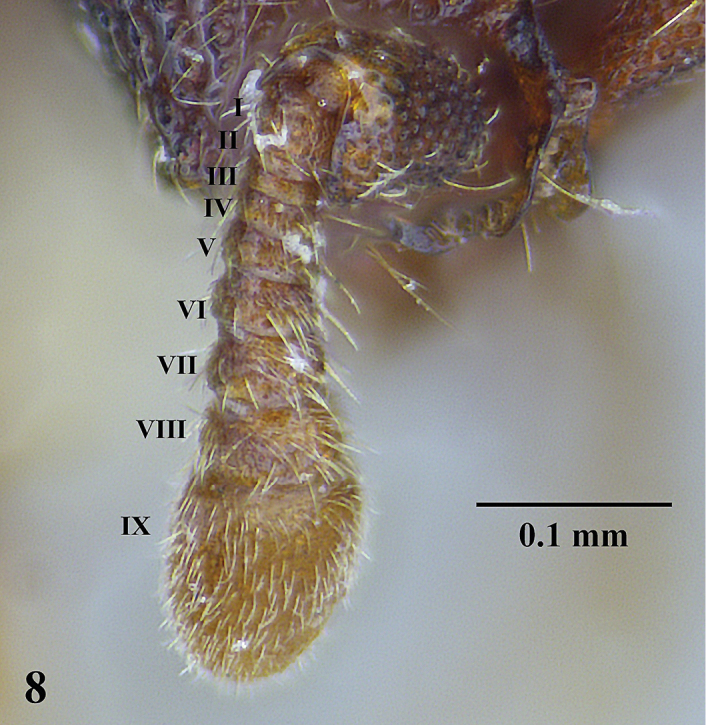
*Ooceraea decamera* sp. nov. Funicular segments.

**Figure 9.**
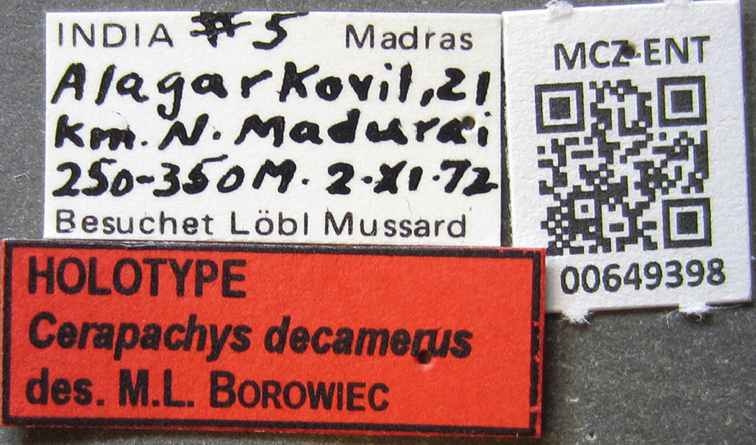
Label of *Ooceraea decamera* sp. nov.

Taxonomic analysis was conducted on a Nikon SMZ 1500 stereo zoom microscope with maximum magnification of 112.5×. Digital images of the specimens were prepared using a Nikon SMZ 1500 stereomicroscope fitted with a Micro Publisher digital camera (Figs 1–4) and Leica MZ 16 stereomicroscope with a JVC digital video camera (Figs 5–9). All the images were cleaned with Adobe Photoshop CS5 and Helicon Filter 5. Morphological measurements were recorded in millimeters on a Nikon SMZ 1500 stereomicroscope. Morphological terminology and standard measurements follow Borowiec (2016) and Yamada et al. (2018).

**HL** Head length: maximum length of head capsule in full-face view, measured from transverse line spanning the anterior most point of clypeus to that of posterior most point of head capsule;

**HW** Head width: maximum width of head capsule in full-face view (excluding eyes);

**SL** Scape length: maximum length of antennal scape excluding basal condylar bulb;

**MW** Mesosomal width: maximum width of promesonotum in dorsal view;

**ML** Mesosomal length: maximum diagonal length of mesosoma in lateral view, measured from posterodorsal border of pronotal flange to posterior basal angle of metapleuron;

**PL** Petiolar length: maximum length of petiole in lateral view;

**PH** Petiolar height: maximum height of petiole in lateral view (including subpetiolar process);

**PW** Petiolar width: maximum width of petiole in dorsal view;

**PPL** Postpetiolar length: maximum length of postpetiole in lateral view;

**PPH** Postpetiolar height: maximum height of postpetiole in lateral view;

**PPW** Postpetiolar width: maximum width of postpetiole in dorsal view;

**CI** Cephalic index: HW/HL × 100;

**SI** Scape index: SL/HW × 100;

**PI1** Petiolar index 1: PL/PH × 100;

**PI2** Petiolar index 2: PW/PL × 100;

**PPI1** Postpetiolar index 1: PPL/PPH × 100;

**PPI2** Postpetiolar index 2: PPW/PPL × 100;

**WI** Waist index: PPW/PW × 100.

**Depositories**

**PUAC**Punjabi University Patiala Ant Collection at Department of Zoology and Environmental Sciences, Punjabi University, Patiala, Punjab, India;

**MCZC**Museum of Comparative Zoology, Harvard University, Cambridge, Massachusetts, United States.

## Results

### Ooceraea joshii sp. nov.

Taxon classificationAnimaliaHymenopteraFormicidae

9DCFFE46-DF69-52AC-8558-AA33512A3640

http://zoobank.org/182F8A89-653B-4604-8337-F7A5F258080B

Figs 1 , 2 , 3 , 4

#### Type locality.

India, Kerala, Periyar Tiger Reserve 9.5627°N, 77.2348°E, 780 m.

#### Type material.

***Holotype*** worker and one ***paratype*** worker, both India, Kerala, Periyar Tiger Reserve 9.5627°N, 77.2348°E, 780 m, leaf litter, Winkler, 21 January 2017, Tarun Dhadwal leg. [PUAC].

#### Measurements.

Holotype: HL 0.57; HW 0.56; SL 0.34; MW 0.39; ML 0.68; PL 0.29; PH 0.34; PW 0.27; PPL 0.32; PPH 0.34; PPW 0.30; CI 98; SI, 61; PI1 85; PI2 93; PPI1 94; PPI2 88; WI 111. Paratype: HL 0.57; HW 0.56; SL 0.33; MW 0.39; ML 0.68; PL 0.29; PH 0.33; PW 0.26; PPL 0.32; PPH 0.34; PPW 0.30; CI 98; SI, 59; PI1 88; PI2 89; PPI1 94; PPI2 88; WI 111.

#### Worker description.

Head in full-face view, almost as long as broad, with lateral margin weakly convex and converging anteriorly, with posterior margin concave medially and posterior lateral corners rounded. Anterior clypeal margin reduced and slightly concave in the middle. Eyes present, small in size, with two ommatidia, parafrontal ridge prominently produced. Mandibles edentate, sub-triangular. Antenna 10-segmented; scape short and clavate, reaching almost mid-length of the head; apical funicular segment fusiform. Frontal lobes reduced. Antennal sockets fully exposed from above.

Mesosoma in lateral view weakly convex; promesonotal suture and metanotal groove absent. Pronotum in dorsal view anteriorly marginate. Propodeum in dorsal view with posterior margin concave; propodeal declivity in lateral view slightly concave, with lateral margin slightly marginate; propodeal lobe reduced. Petiolar node in dorsal view as long as broad, rounded anteriorly, in lateral view hemiglobular; subpetiolar process well-developed, with sickle-shaped anteroventral apex. Postpetiole in dorsal view subtrapezoidal, with anterior margin transverse and posterior margin convex, in lateral view with anteroventral corner angulate. Gastral segment I (abdominal segment IV) large, occupying the most part of gaster, in lateral view with dorsal margin weakly and roundly convex.

Sculpture. Head foveolate-reticulate; mesosoma, petiole and postpetiole foveolate-reticulate; gaster foveolate, with foveae smaller than those of head and mesosoma.

Pilosity and Pubescence. Body covered with erect or sub-erect hairs; sides of head and legs covered with shorter hairs; scape and funicular segments covered with short decumbent or subdecumbent hairs.

Body coloration. Head and gaster light brown; mesosoma, petiole and postpetiole darker than the head; legs yellowish brown.

**Queen.** Unknown.

**Male.** Unknown.

#### Recognition.

*Ooceraea joshii* sp. nov. and *O. decamera* sp. nov. (described below) are distinctly separated from the other valid congeners by having 10-segmented antennae. Furthermore, the two new species are well distinguished from each other by a combination of the following characters: head shape (almost as long as broad in *O. joshii* sp. nov., rectangular, distinctly longer than broad in *O. decamera* sp. nov.); presence of ommatidia (present in *O. joshii* sp. nov. and absent in *O. decamera* sp. nov.); propodeal lobes (reduced versus roundly produced); petiolar node in lateral view (hemiglobular versus rectangular); subpetiolar process (anteroventral part sickle-shaped versus forming a rectangular and semitransparent lobe); pilosity (head and body comparatively more pilose in *O. joshii* sp. nov.); and sculpturation (head, mesosoma, petiolar, postpetiolar node, and gaster with more pronounced foveolate sculpture in *O. joshii* sp. nov.).

#### Bionomics.

The type series was found in leaf litter samples collected from the Medaganam region of the Periyar Tiger Reserve situated at an elevation of 780 meters. The region is composed of an undisturbed tropical moist evergreen forest with low light penetration, with a mean average daytime temperature of 30 °C.

#### Distribution.

Known only from the type locality.

#### Etymology.

The species has been named in honor of Professor Amitabh Joshi, a distinguished evolutionary biologist based at Jawaharlal Nehru Centre for Advanced Scientific Research (JNCASR), Bengaluru, India.

### Ooceraea decamera sp. nov.

Taxon classificationAnimaliaHymenopteraFormicidae

EFC710C7-7193-5101-BA40-0E0AD282BD0C

http://zoobank.org/D8C9E609-7416-4081-A54A-83CAA01CEAD6

Figs 5 , 6 , 7 , 8 , 9

#### Type locality.

India: Madras, Alagarkovil, 21 km. N Madurai, 10.02308°N, 77.833333°E, 250–350 m alt.

#### Type material.

***Holotype*** worker, India, Madras, Alagarkovil, 21 km N Madurai, 10.02308°N, 77.833333°E, 250–350 m alt.; 2 November 1972; Besuchet Lobt Mussard leg. (Specimen number/barcode: MCZ-ENT00649398) [MCZC].

#### Holotype measurements.

HL 0.62; HW 0.46; SL 0.26; MW 0.38; ML 0.78; PL 0.26; PH 0.42; PW 0.30; PPL 0.34; PPH 0.41; PPW 0.40; CI 74; SI 57; PI1 62; PI2 93; PPI1 81; PPI2 118; WI 133.

#### Worker description.

Head in full-face view rectangular, distinctly longer than broad (CI 74), with lateral sides weakly convex, with posterior margin concave medially, with occipital lobes/corners angulate. Anterior clypeal margin slightly projecting forward. Eyes absent. Parafrontal ridge prominent and elevated. Mandibles edentate but weakly serrate. Antennae with 10 segments; scape short, clavate, slightly surpassing the mid-length of head. Frontal lobes reduced. Antennal sockets fully exposed from above.

Mesosoma in lateral view almost flat; promesonotal suture and metanotal groove absent. Pronotum in dorsal view with anterior margin weakly and roundly convex, with humeral corner rounded. Propodeum in dorsal view with posterior margin concave; propodeal declivity in lateral view almost straight, with lateral margin marginate; propodeal lobe roundly produced. Petiolar node in dorsal view subtrapezoidal, distinctly broader than long, in lateral view rectangular with anterior and posterior margins almost straight and dorsal margin weakly convex. Subpetiolar process well-developed, with anteroventral part forming a rectangular and semitransparent lobe. Postpetiole broader than long, anterior margin weakly concave and posterior margin weakly convex, in lateral view with anteroventral part broadly and roundly produced. Gastral segment I (abdominal segment IV) large occupying the most part of gaster, in lateral view with dorsal margin almost straight, base of cinctus of first gastral tergite cross-ribbed.

Sculpture. Head, mesosoma, petiole and postpetiole shallowly foveolate-reticulate; mandibles and dorsal surface of gaster sparsely foveolate, foveae somewhat smaller as compared to those present on head, mesosoma, petiole, and postpetiole.

Pilosity and pubescence. Whole body covered with pale yellow erect and sub-erect hairs; appressed pubescence abundant on antennae and legs.

Body coloration. Mandibles, antennae, legs, subpetiolar process and gaster light brown; head, mesosoma and gaster dark brown.

**Queen.** Unknown.

**Male.** Unknown.

#### Recognition.

The two species significantly differ from each other on the basis of dimensions of head capsule and shape of subpetiolar process.

#### Bionomics.

Unknown.

#### Distribution.

Known only from the type locality. The place has been transformed into agricultural land and is prone to anthropogenic activities. Thus, this reinforces the concept that important biodiversity components, which are already rare, are imperiled due to local extinctions.

#### Etymology.

The species epithet *decamera* refers to the ten-segmented antennal count.

### Illustrated key to the known species of *Ooceraea* based on worker caste

**Table:** 

1	Whole body variously sculptured	**2**
–	Whole body not sculptured, mesosoma smooth and shiny (Fig. A)	***O. australis***
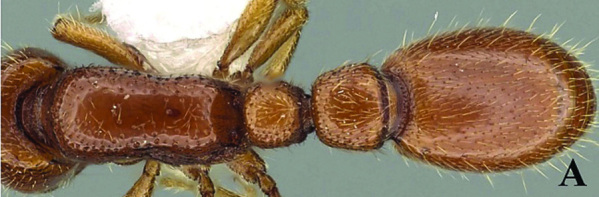		
2	Body predominantly punctate (Fig. A)	**3**
–	Body predominantly foveate (Fig. B)	**6**
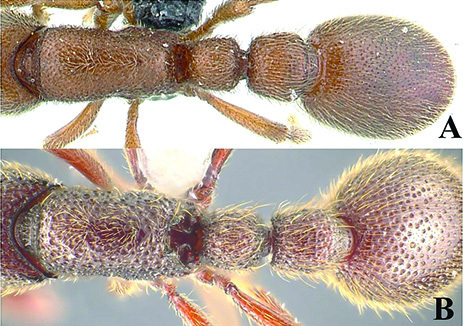		
3	Anterolateral shoulders of the first gastric segment as seen from above broadly rounded and gradually widening caudad (Fig. A)	***O. biroi***
–	Anterolateral shoulders of the first gastric segment abruptly rounded, accentuating the medium concavity that receives the postpetiole (Fig. B)	**4**
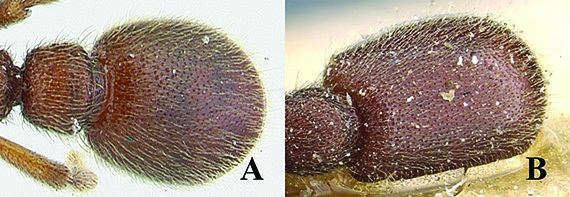		
4	Postpetiole distinctly longer than broad (Fig. A)	**5**
–	Postpetiole broader than long (New Guinea) (Fig. B)	***O. papuana***
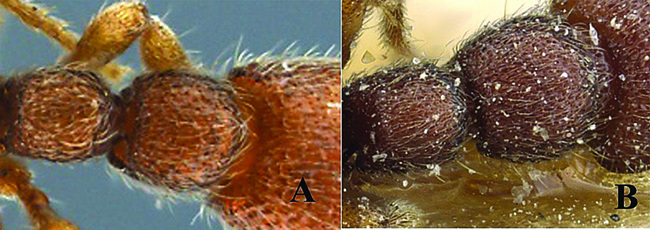		
5	Head coarsely and irregularly rugose and punctuate (Solomon Islands)	***O. pawa***
–	Head regularly punctate (New Guinea) (Fig. A)	***O. pusilla***
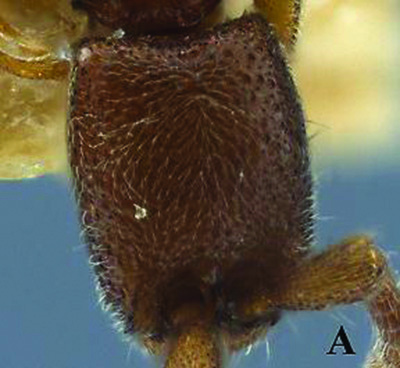		
6	Lateral ridge of posterior face of propodeum armed with two pairs of denticles (Vietnam) (Fig. A)	***O. quadridentata***
–	Lateral ridge of posterior face of propodeum without denticles (Fig. B)	**7**
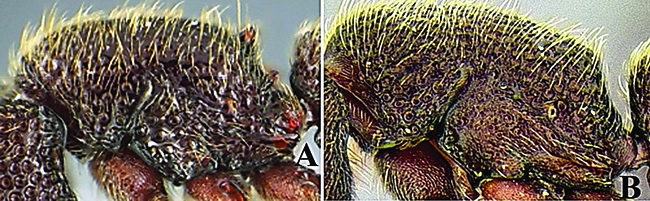		
7	Spaces between foveolae shagreen-like, giving the integument a rough appearance (Fig. A)	**8**
–	Spaces between foveolae smooth, giving the integument a shiny appearance (Fig. B)	**10**
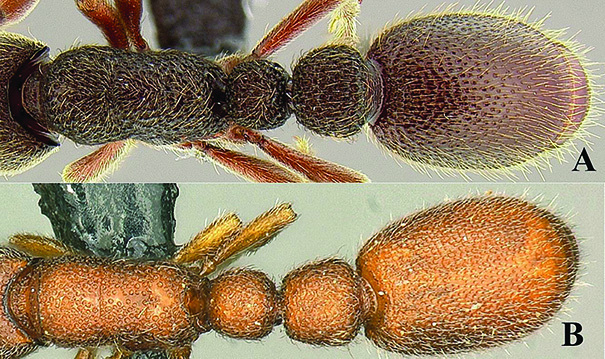		
8	Species large in size (HW> 70mm); posterior margin of head almost straight (Fig. A)	**9**
–	Species small in size (HW< 40 mm); posterior margin of head distinctly concave in the middle (Fig. B)	***O. alii***
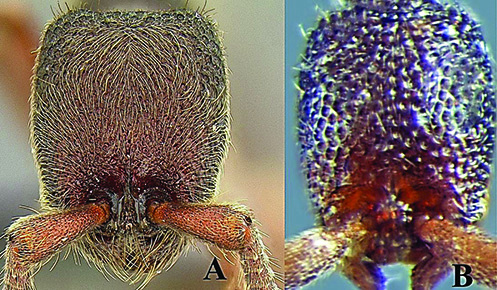		
9	Sides of propodeum and sides of petiole with dense, deeply impressed foveae that are discernibly wider in diameter than propodeal spiracle or foveae on head. Dark species (Fiji) (Fig. A)	***O. fuscior***
–	Sides of propodeum and sides of petiole with sparse, lightly impressed foveae that are not discernibly wider in diameter than propodeal spiracle or foveae on head. Pale species (Fiji) (Fig. B)	***O. crypta***
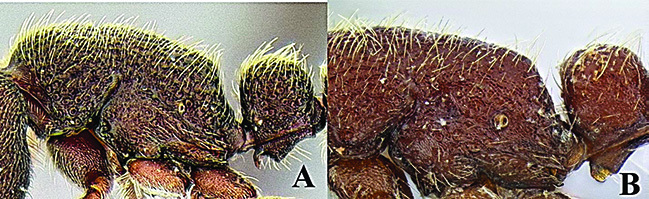		
10	Antenna 8-segmented (China)	***O. octoantenna***
–	Antenna more than 8-segmented	**11**
11	Antenna 10-segmented	**12**
–	Antenna 11-segmented	**13**
12	Head as long as broad; eyes present; propodeal lobes reduced; petiolar node in lateral view hemiglobular; anteroventral part of subpetiolar process sickle-shaped; head and body comparatively more pilose (India) (Fig. A, B)	***O. joshii* sp. nov.**
–	Head distinctly longer than broad; eyes absent; propodeal lobes roundly produced; petiole node in lateral view rectangular; anteroventral part of subpetiolar process forming a rectangular and semitransparent lobe (India) (Fig. C, D)	***O. decamera* sp. nov.**
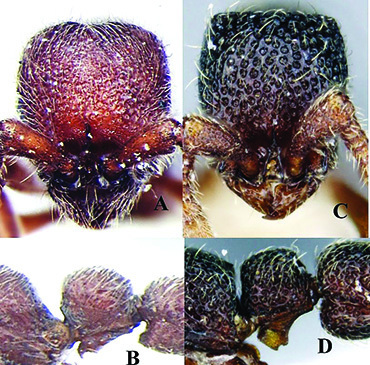		
13	Body shallowly and coarsely foveolate; eye present with multiple ommatidia (India) (Fig. A)	***O. besucheti***
–	Body conspicuously foveolate; eye absent or vestigial (Fig. B)	**14**
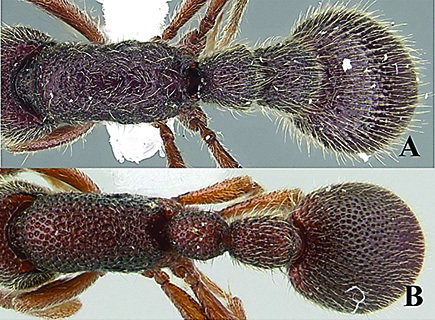		
14	Anterior portion of pronotum with distinct ridge; petiole and postpetiole in dorsal view broader than long (Sri Lanka) (Fig. A)	***O. coeca***
–	Anterior portion of pronotum without distinct ridge; petiole and postpetiole in dorsal view longer than broad (Sri Lanka) (Fig. B)	***O. fragosa*** ^1^
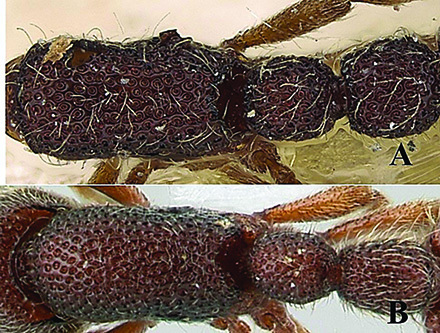		

**Figure 10.**
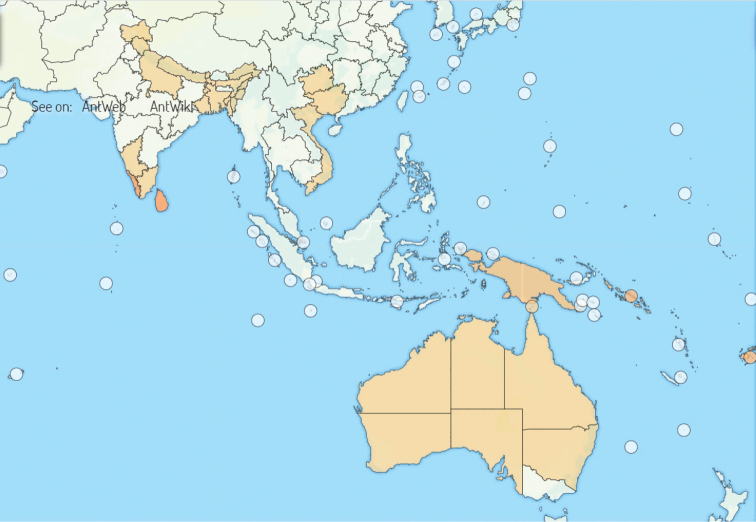
Map showing the current distribution of *Ooceraea* species.

**Table 1.** Antennal count and distribution of *Ooceraea* species in different biogeographical regions.

Sr. No.	Species	Antennal count	Biogeographical region
1.	*Ooceraea octoantenna* Zhou et al., 2020	8	Palearctic
2.	*Ooceraea alii* (Bharti & Akbar, 2013)	9	Oriental
3.	*Ooceraea australis* (Forel, 1900)	9	Australasia
4.	*Ooceraea biroi* (Forel, 1907)	9	Malagasy, Neotropical, Oceania, Oriental, Palearctic
5.	*Ooceraea crypta* (Mann, 1921)	9	Oceania
6.	*Ooceraea fuscior* (Mann, 1921)	9	Oceania
7.	*Ooceraea papuana* Emery, 1897	9	Australasia
8.	*Ooceraea pawa* (Mann, 1919)	9	Australasia
9.	*Ooceraea pusilla* Emery, 1897	9	Australasia
10.	*Ooceraea quadridentata* Yamada et al., 2018	11	Oriental
11.	*Ooceraea besucheti* (Brown, 1975)	11	Oriental
12.	*Ooceraea coeca* Mayr, 1897	11	Oriental
13.	*Ooceraea fragosa* Roger, 1862	11	Oriental
14.	*Ooceraea guizhouensis* (Zhou, 2006)	11	Palearctic

## Supplementary Material

XML Treatment for Ooceraea joshii

XML Treatment for Ooceraea decamera

## Footnotes

1 Note: Comprehensive morphological diagnosis and image of O. guizhouensis (Zhou, 2006) is not available, so this species has been not included in the key. However, as cited in the comparative diagnosis, O. guizhouensis is close to O. besucheti and O. fragosa, but differs in having coarse pilose punctures on the surface of the mandibles, petiolar node distinctly longer than broad and is without eyes.
